# Timing surgery in mitral regurgitation: defining risk and optimising intervention using stress echocardiography

**DOI:** 10.1530/ERP-16-0019

**Published:** 2016-12

**Authors:** Boyang Liu, Nicola C Edwards, Simon Ray, Richard P Steeds

**Affiliations:** 1 University Hospital Birmingham NHS Foundation Trust & Institute of Cardiovascular Sciences, University of Birmingham, Birmingham, UK; 2 University Hospital South Manchester, Manchester, UK

**Keywords:** stress echocardiography, mitral valve repair, mitral valve replacement, mitral regurgitation, timing of surgery

## Abstract

Mitral regurgitation (MR) is the second most common form of valvular disease requiring surgery. Correct identification of surgical candidates and optimising the timing of surgery are key in management. For primary MR, this relies upon a balance between the peri-operative risks and rates of successful repair in patients undergoing early surgery when asymptomatic with the potential risk of irreversible left ventricular dysfunction if intervention is performed too late. For secondary MR, recognition that this is a highly dynamic condition where MR severity may change is key, although data on outcomes in determining whether concomitant valve intervention is performed with revascularisation has raised questions regarding timing of surgery. There has been substantial interest in the use of stress echocardiography to risk stratify patients in mitral regurgitation. This article reviews the role of stress echocardiography in both primary and secondary mitral regurgitation and discusses how this can help clinicians tackle the challenges of this prevalent condition.

## Introduction

Mitral regurgitation (MR) is the second most common type of valve disease requiring surgery in Europe (1). Despite a reduction in the incidence of rheumatic heart disease, the frequency of MR is increasing due to an ageing population (2). Improvements in the diagnosis, quantification and operative techniques for mitral valve (MV) repair now allow the restoration of normal life expectancy after surgery (3). The timing and type of surgery depend upon a number of factors, one of the most important being whether the MR is primary or secondary (Videos 1 and 2). The balance in each case then lies between facing the risks of early surgery and risking left ventricular (LV) dysfunction if intervention is performed too late. In addition, consideration has to be given to the risk of mitral valve replacement in case of immediate failure of repair and the risk of re-intervention in the situation of late failure of repair. Unfortunately, a significant proportion of those with severe MR who have apparently normal LV function pre-operatively (LVEF >60%) continue to present post-operatively with reduced ejection fraction and congestive cardiac failure (4). This article outlines the discussion surrounding the timing of surgery and highlights the importance of exercise stress echocardiography in the management of primary and secondary MR.

Video 1 Primary mitral regurgitation with flail A2 scallop of the anterior mitral valve leaflet. View Video 1 at http://movie-usa.glencoesoftware.com/video/10.1530/ERP-16-0019/video-1.
Download Video 1

Video 2 Secondary ischaemic mitral regurgitation (asymmetric). View Video 2 at http://movie-usa.glencoesoftware.com/video/10.1530/ERP-16-0019/video-2.
Download Video 2

## Controversy in the timing of surgery for primary MR

Class 1 indications for surgery in primary MR have been unchanged for many years (5). In recent years, this ‘conventional’ approach has been challenged by advocates for early MV repair who have come to regard standard class I triggers such as heart failure and LV dysfunction as promoting ‘rescue surgery’ (6). Data to support this view originated in operative series from the 1980s, which highlighted improved surgical outcomes in patients operated with NYHA class I–II rather than NYHA III–IV symptoms (3). In the modern era, several observational series have consistently demonstrated adverse outcomes for each of the individual components of the current class I surgical indications. In a prospective surgical series of 840 patients with MR amenable to repair, worsening NYHA classification was associated with a stepwise reduction in late survival rates 20 years after surgery (7). In a retrospective case registry, mortality was increased by 80% at 10-year follow-up once EF had fallen to 50–59% compared with those in whom LVEF remained above 60% (8). Furthermore, LV dilatation above 40 mm in end-systole in primary MR predicted excess mortality and was an independent predictor of permanent post op LV dysfunction (9). Summarising the risks of delay to repair, a retrospective of 1512 patients undergoing isolated primary MR at the Rochester Mayo clinic between 1990 and 2000 found long-term survival at 15 years was only 42 ± 2% for patients with a class I indication, compared with 53 ± 4% for those with a class II surgical indication including presence of atrial fibrillation or pulmonary hypertension, with the highest survival (70 ± 3%) observed in subjects receiving early operation based on the presence of a high probability of successful repair. Furthermore, operative mortality was only documented in those with a class I indication for surgery (6). These data have led authors to suggest that current class 1 recommendations are criteria that do not promote optimal outcomes for patients with severe primary MR.

## Outcomes of mitral repair in primary MR

Registry studies have established that the results of MV repair are superior to replacement, even in the elderly (10). Prosthetic valve replacement is associated with higher operative mortality, reduced life expectancy, higher long-term risk of stroke and complications specific to valve replacement such as valve thrombosis and structural valve degeneration (11). If repair can be successfully performed before the onset of advanced symptoms, data from expert centres report equivalent long-term outcomes to age- and gender-matched controls at >20-year follow-up (7). Both prospective and registry data support early repair before class 1 indications are reached. A single-centre prospective study of 610 consecutive patients with asymptomatic severe MR diagnosed with quantitative echocardiography compared outcomes between those referred for early surgery (235 patients; 94% repair rate) and those referred with conventional class 1 indications (375 patients; 82% repair rate), with the decision made at the discretion of the referrer. At a follow-up of 12 years, the early surgery group had significantly lower cardiac mortality (HR 0.109; 95% confidence interval (CI) 0.014–0.836; *P* = 0.033) and cardiac event rates (HR 0.216; 95% CI 0.083–0.558; *P* = 0.002) (12). The multi-centre, multi-national Mitral Regurgitation International Database included 2097 consecutive patients with primary MR due to flail segments and found improved survival at 10 years with lower rates of heart failure for those proceeding with early mitral repair compared with those managed medically until class 1 guidelines were triggered (13).

## Mitral repair as a class 2A indication for surgery

Given the consistency of these data, why do current guidelines (5) not emphasise the importance of earlier repair for all patients with severe, degenerative MR? First, the data supporting early surgery are mostly from single-centre, non-randomised studies and many findings are from retrospective registries. These have tended to be high-volume centres with specialised, experienced surgeons performing large numbers of MV repairs. Such data on rates of repair and lower perioperative morbidity and mortality cannot always be extrapolated to lower-volume centres (14, 15). Randomised prospective studies in mixed populations are lacking; however, trials are under way (16). Secondly, the benefits of such long-term outcomes are mainly reserved for those with primary degenerative disease (Carpentier mechanism type II) and outcomes are less consistent for other causes, including rheumatic disease (17). Thirdly, it can be more difficult to persuade an asymptomatic patient in clinic to undergo major cardiothoracic surgery – by definition, prophylactic surgery in asymptomatic individuals does not improve how they feel (although this is not an issue in conditions when mortality benefit is clear, for example aortic aneurysm surgery). Finally, there are also data that suggest careful outpatient care may deliver outcomes that are as good. In a study of 132 consecutive patients with asymptomatic severe MR, a programme of annual review with referral based on class 1 indications also delivered outcomes equivalent to the general population over a follow-up period of 62 ± 26 months – but with the added advantage that 55 ± 6% of the population were able to avoid surgery completely at 8-year follow-up without complication (18). Of equal importance was that surgical outcomes were also excellent, with no compromise in symptomatic status or LV outcome from delay.

Moreover, referral for early surgery may often not be a straightforward decision – even with quantitative echocardiography, grading severe MR is subject to significant variation between operators (19). When severe MR is confirmed, the ability to identify a reparable MV is not perfect. Although in the US there has been a significant improvement in rate of repair with promotion of the ‘mitral valve surgeon’ and discouragement of lower volume centres so that repair is performed in excess of 90% cases (20), this is not universal across all surgeons and in all countries. In the randomised prospective study of Kang and coworkers, recruiting only those with presumed reparable valves, repair was actually carried out in 94% (12). In the UK, audit data from the National Institute for Cardiovascular Outcomes Research (NICOR) identified that 1558 isolated first-time MV repairs were carried out in 2013, compared with 789 isolated MV replacements. The 1-year and 5-year mortality rates for isolated MV repair in 2013 were 1.1% and 11% respectively, but double for MV replacement at 2.4% and 20% (21). Such diverse surgical practice is of major concern, especially when considering the higher mortality and morbidity with MV replacement compared with MV repair and has prompted a call for defined centres of excellence for mitral valve repair in the UK with the presence of a dedicated heart valve team (22). Finally, MV repair is not risk free. In the UK, NICOR data reported mortality in 2013 for the first time, isolated MV repair at 1.09% and 2.79% when combined with coronary artery bypass grafting (CABG) (21). Furthermore, although actual survival far exceeds expected outcomes (5.19%), MR can still recur after repair. In the most recent series, rates of recurrent MR were 13.3 + 1.2% patients at 15-year follow-up, with a reoperation rate of 6.9 + 1.0% (23). Although serial data from this study suggest that rates of late failure are falling, possibly as a result of technical improvements such as routine use of ring annuloplasty and peri-operative 3D transoesophageal echocardiography, recurrence of MR after MV repair is associated with adverse LV remodelling and increased risk of death (23). For an asymptomatic patient to run this risk, there has to be certainty for that person that his or her own operative risk is low, that valve repair will be durable and that their own life expectancy will be long enough to benefit in the long term from any prognostic gain.

## Stress echocardiography: improving risk stratification in primary MR

In addition to markers of adverse outcome that include the onset of atrial fibrillation (24), pulmonary hypertension (6) and left atrial dilatation (25), objective testing of symptom status is a critical step in decision making. Studies in those with severe aortic stenosis have emphasised that patients often minimise their symptoms by avoiding exercise and that objective testing may reveal limitations unsuspected on history alone. Similarly, in severe ‘asymptomatic’ MR, 20% have a sub-maximal functional capacity on cardiopulmonary exercise testing (26). Event-free survival is lower in those asymptomatic patients with severe primary MR who have a reduced exercise capacity despite good LV function and normal LV dimensions (27). In a large study of 884 consecutive patients undergoing exercise stress echocardiography, exercise capacity (lower than 100% age/sex predicted metabolic equivalents (METs) achieved), heart rate recovery (HRR, <18 beats within 1 min after exercise) after stress- and exercise-induced atrial fibrillation were strong independent markers of adverse outcome in primary MR (28). These data emphasise the importance of monitoring apparently asymptomatic patients using a regular exercise test to provide objective assessment of symptom status. In those with no exercise limitation according to age- and gender-based predicted metabolic equivalents, delay in surgery does not impair outcome at one year (29).

**Figure 1:**
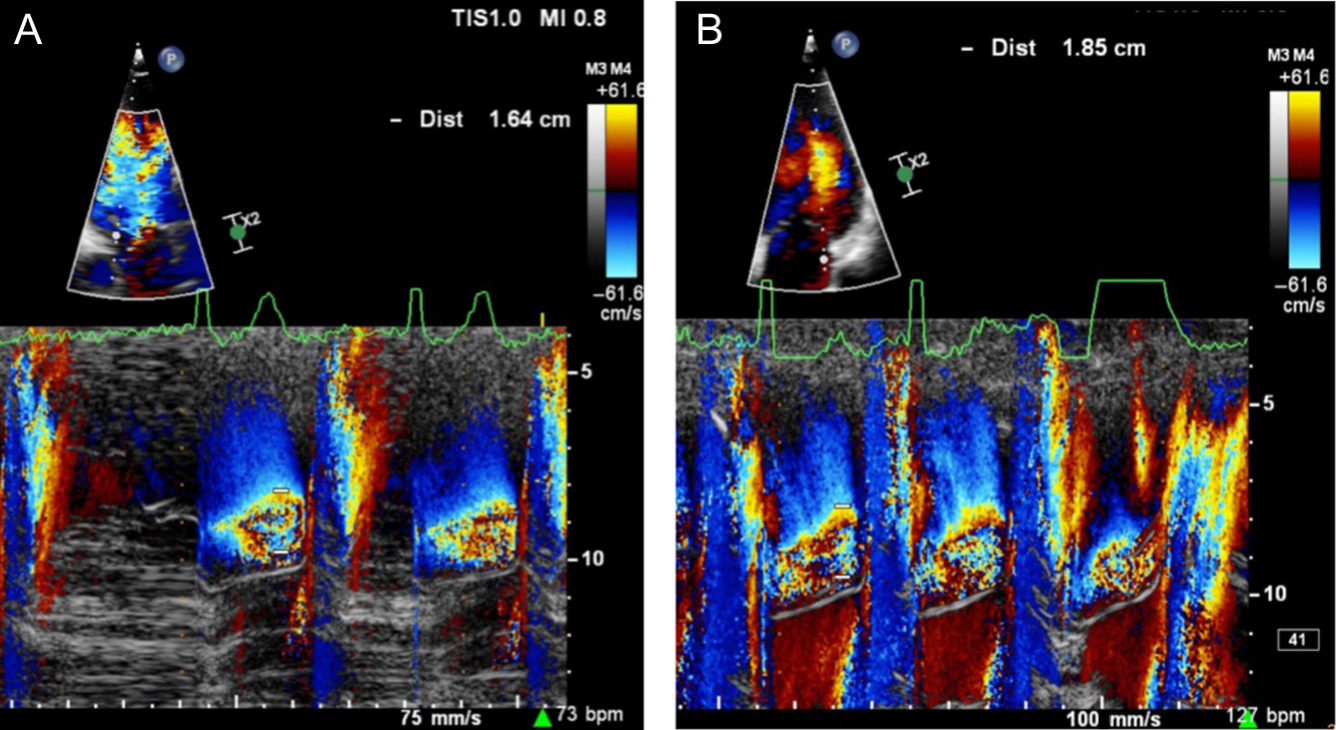
Colour M-mode demonstrating worsening MR in a patient with normal LVEF. There is an increase in PISA from rest (A) to exercise (B) while cycling at 75 W.

**Figure 2:**
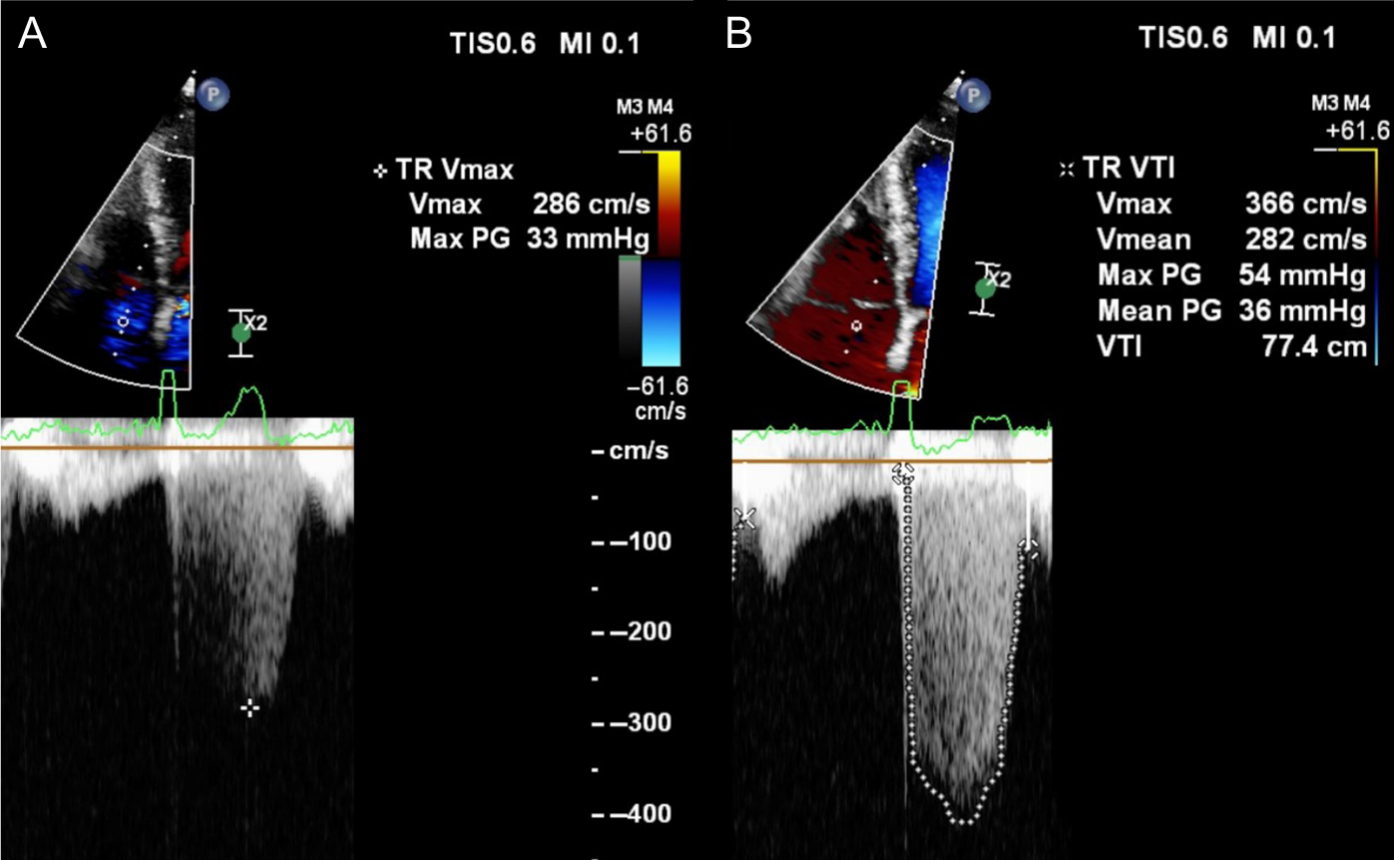
Continuous wave Doppler demonstrating an increase in severity of tricuspid regurgitation and increase in maximal velocity from rest (A) to exercise (B) while cycling at 75 W.

**Figure 3:**
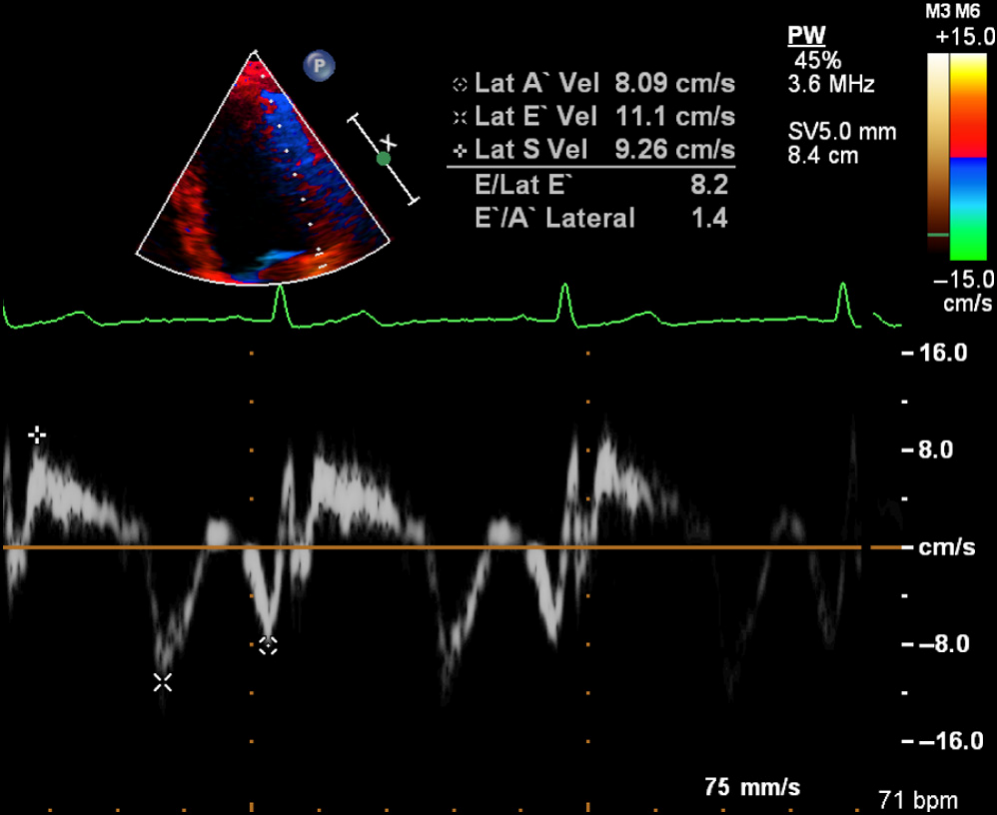
Example of latent contractile dysfunction with normal LVEF (Video 3), but systolic tissue velocity below 10.5 cm/s at rest.

If echocardiography is added to the exercise test, what further information can be gleaned? First, a change in severity of primary MR with exercise is common and occurs in over 30% of those with asymptomatic moderate-to-severe MR (Fig. 1A and B) (30). From a small study of 61 asymptomatic patients, those who have an increase in effective regurgitant orifice area (EROA) of more than 10 mm^2^ during exercise have a lower symptom-free survival compared with those with no change. Secondly, patients with moderate-to-severe MR who develop pulmonary hypertension above 60 mmHg on exercise are at greater risk of symptoms and adverse outcomes (Fig. 2A and B) (30, 31). Thirdly, an assessment of LV function during stress echocardiography can also be an important marker of post-operative outcome. The onset of symptoms is not only governed by severity of MR and its effect on pulmonary pressure but also on the capacity of the LV to respond to volume-loading. Resting LV ejection fraction can be a poor marker of myocardial contractility as over a third of patients with a ‘normal’ pre-operative EF >60%, develop LV dysfunction below 50% after successful mitral repair (4). Latent contractile dysfunction can be predicted by measuring a systolic tissue velocity (<10.5 cm/s at rest) (Video 3 and Fig. 3) (32) and by quantifying global longitudinal strain (<−20%) (33). Improved outcomes can also be predicted by the LV response to exercise. In 71 consecutive asymptomatic patients with isolated moderate-to-severe primary MR, those in whom the LVEF failed to increase by ≥4%, had poorer symptom-free survival and worse outcomes after MV surgery (34). Similarly, a failure in global longitudinal strain to improve by ≥2% with exercise (Videos 4 and 5) appears to be a more sensitive marker of latent contractile dysfunction when indexing strain to end-systolic volume (35).

Video 3 Example of latent contractile dysfunction with normal LVEF but systolic tissue velocity below 10.5 cm/s at rest (Fig. 3). View Video 3 at http://movie-usa.glencoesoftware.com/video/10.1530/ERP-16-0019/video-3.
Download Video 3

Video 4 Example of reduction in GLS from rest in a patient with mitral regurgitation and LVEF >60%. View Video 4 at http://movie-usa.glencoesoftware.com/video/10.1530/ERP-16-0019/video-4.
Download Video 4

Video 5 Example of reduction in GLS to exercise in a patient with mitral regurgitation and LVEF >60%. View Video 5 at http://movie-usa.glencoesoftware.com/video/10.1530/ERP-16-0019/video-5.
Download Video 5

## Why does primary MR worsen during exercise?

**Figure 4:**
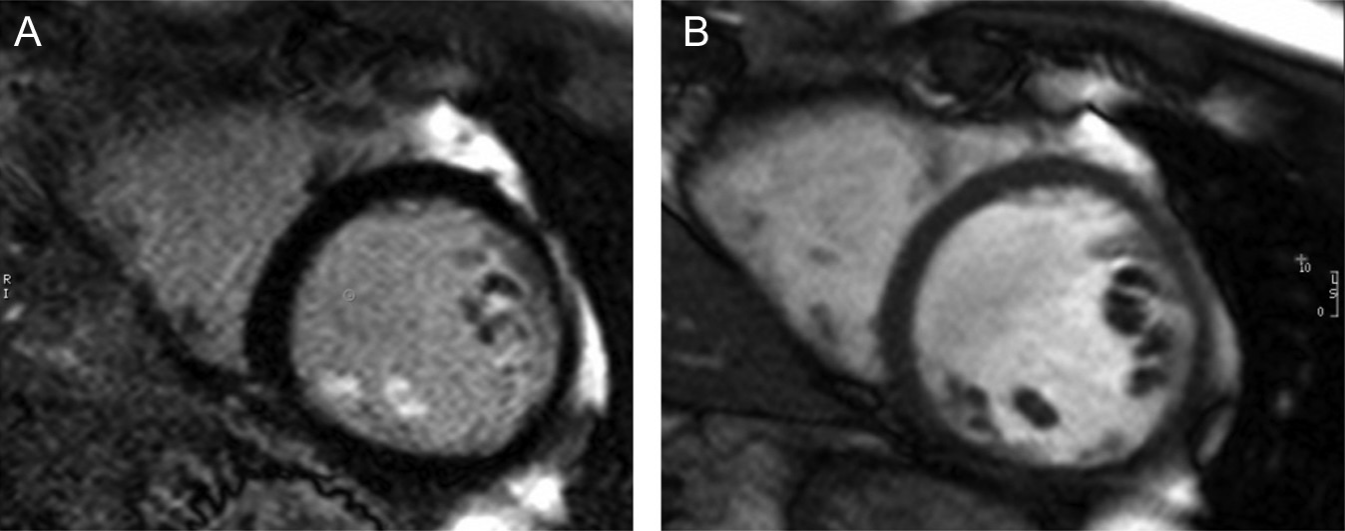
Late gadolinium enhancement of papillary muscle on cardiac MRI (A) and the corresponding cine short axis slice (B) in a patient with primary degenerative MR and normal LVEF.

It has been suggested that exercise promotes an increase in systolic blood pressure, but pressure increase alone should not have a major impact on regurgitation without increase in regurgitant orifice as well as flow varies only with the square root of the pressure change between the LV and atrium (30). It is more likely that this may be due to changes in LV and annular geometry (36). Exercise-associated reduction in end-systolic volume could redefine the relationship between the papillary muscles and the zone of apposition of the leaflets (37). A further possibility is that the repetitive prolapse of a degenerate MV leads to papillary muscle traction, resulting in the fibrosis that has been documented on late enhancement with cardiovascular magnetic resonance imaging (Fig. 4) (38). Fibrosis in turn could promote a failure of the papillary muscles to respond to exercise, resulting in additional MR, a possibility suggested by differences in papillary muscle velocity and excursion in those with prolapse (39). These factors require further investigation as an understanding of the mechanisms of exercise-related MR may in turn further define those valves that need early repair.

## Current indicators for stress echocardiography in primary MR

Current US guidelines support the use of exercise testing in the assessment of patients with asymptomatic severe valvular heart disease to help identify those with latent symptoms and to clarify prognosis (40). European guidelines support the addition of echocardiography to exercise stress testing in experienced hands for the assessment of patients whose symptoms or LV dysfunction appear disproportionate to the severity of MR at rest (5, 41). In the future, use of stress may be more widespread because it is possible that careful assessment of the patient with asymptomatic severe MR with normal cavity dimensions and good LV function may define two groups of patients – (1) those who have a good prognosis who can avoid surgery despite having a repairable valve and (2) those who are more likely to progress to symptoms and LV dysfunction who need surgery earlier. Unfortunately, there are as yet no randomised data that compare the outcome of such a strategy.

## Secondary MR

Secondary MR is a dynamic condition where the degree of MR at rest does not predict the degree of MR on exertion. In a study of 70 consecutive patients with ischaemic LV dysfunction, MR decreased in 13 (19%), increased by less than 13 mm^2^ when measured by effective regurgitant orifice area (EROA) in 38 (54%) and increased more than this in the remaining 19 patients (42). The primary determinants of exercise-related deterioration in MR appear to be systolic annular area, degree of tenting of the valve and the associated wall motion abnormalities (42). These are dependent on the extent of ischaemic damage, exercise-induced dyssynchrony and the presence or absence of viability within the myocardium and papillary muscles; indeed, the severity of MR may reduce in patients with viable myocardium due to myocardial recruitment (43, 44, 45). Whether secondary MR improves or deteriorates on exercise is important as an increase in severity of MR with exercise by EROA ≥13 mm^2^ is associated with a five-fold increased risk of subsequent cardiac death (46). These and other data have led to support for the role of stress echocardiography in the investigation of patients with shortness of breath on exertion but who have less than severe secondary MR at rest. As yet, however, the role of stress echocardiography in the timing of mitral valve surgery in secondary MR remains controversial. In part, this is due to the lack of data, but in addition, it is not yet clear how best to manage patients with secondary MR.

## Timing of surgery in chronic secondary MR

Secondary (ischaemic) MR is usually defined as the presence of MR either at rest or on exertion present more than 2 weeks after myocardial infarction. Although the frequency with which MR is detected may vary according to the method of imaging and the time at which this is carried out after MI, the presence of secondary MR confers a graded inverse relationship with risk of cardiovascular deaths (RR 1.88, 95% CI 1.23–2.86). Even those with mild ischaemic MR have a significantly worse survival (47). Although either the presence of MR and/or deterioration after exercise identifies a group of patients at particularly high risk, there continues to be controversy over whether surgical correction improves either life expectancy or quality of life (48). MV repair plus CABG was found to improve NYHA class, oxygen consumption on exercise testing and end-systolic volume index compared with CABG alone in a multi-centre study randomising 73 moderate MR patients, although mortality rates were similar in both groups (49). A similar finding was noted in a trial of 102 patients with an improvement in NYHA class after combined CABG and MV repair (50). In contrast, a further randomised study of 301 patients did not demonstrate concomitant MV repair led to any improvements in LV remodelling at 2-year follow-up but instead was associated with longer bypass time, hospital stay and more neurological events (48, 51). There is also controversy over whether MV repair or MV replacement produces the better outcome, with no apparent difference in end-systolic volume or mortality between approaches but less recurrent MR with replacement (52). These studies have generated a debate on whether secondary MR is only a marker of poor LV function rather than independently contributing to adverse outcome (53). Despite the inconsistency of evidence, it remains a class 2A indication to include MV repair at the time of CABG if MR is severe, whereas repair or replacement may be considered only if patients are severely symptomatic despite optimal medical therapy (class 2B) (40).

## Stress echocardiography in secondary MR?

Although secondary MR may vary during exercise and diagnosis is associated with outcome, the question arises as to whether stress echocardiography could play a role in clarifying treatment strategy in symptomatic patients. In clinical practice, the main potential appears to be in two groups of patients. First, there are those whose symptoms of breathlessness appear to be disproportionate to the extent of LV impairment or the severity of MR. Exercise echocardiography can be used both to confirm objectively the extent of physical limitation (54), together with an imaging strategy that can define the extent of resting LV impairment, presence of ischaemia, development of dyssynchrony and alteration in the degree of MR during stress (5, 40, 42, 43). Secondly, the degree of secondary MR that is considered significant is relatively minor (EROA ≤13 mm^2^). In those in whom there is discussion as to whether percutaneous or surgical revascularisation should occur, demonstration of a significant exercise-related change in severity of MR or pulmonary hypertension would perhaps support surgery in those with symptoms (55). The limitation once again is that this diagnostic and intervention strategy is based on single-centre, non-randomised studies and large scale, randomised data are needed.

## Performing stress echocardiography in mitral regurgitation

Stress echocardiography in MR should be preceded by a full transthoracic echocardiogram. This baseline echocardiogram should include an assessment of LV volumes and function, wall motion, aortic root, assessment of right ventricular dimensions and function, pulmonary pressure and assessment of all valves.

**Figure 5:**
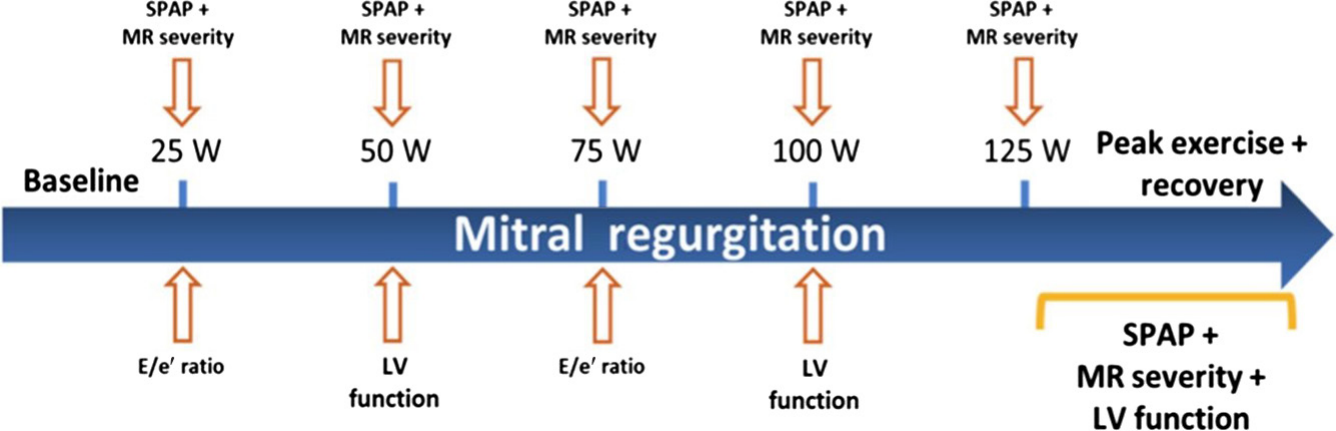
Mitral regurgitation exercise echocardiogram protocol. Reproduced, with permission, from Lancellotti P & Magne J, 2013, Stress echocardiography in regurgitant valve disease, *Circulation: Cardiovascular Imaging*, volume 6, pages 840–849 (56). Copyright 2013 Wolters Kluwer Health Inc.

Most published studies have used upright or semi-supine bicycle exercise as the stressor because this permits continuous imaging at all stages of exercise. MR tends to resolve rapidly with rest, so that although treadmill exercise is useful for assessing symptoms and ischaemia, it is less so for quantifying change in MR. Moreover, bicycle stress provides more isometric stress than aerobic exercise, which may be more useful in evaluating MR. Usually, patients are asked to maintain a cadence of around 60/min with increments in workload of 25 W made at 2-min intervals, although protocols can be altered to younger patients using higher load (56). Data to be acquired are listed in Table 1 with a standard MR exercise echocardiography protocol shown in Fig. 5, illustrating the typical sequence of data acquisition. This generic protocol can of course be modified if there is a specific clinical question – such as more emphasis on LV function if there is an interest in LV viability. It is also useful to be aware of some of the limitations of quantitative echocardiography in MR, specifically the proximal isovelocity surface area (PISA) method for calculation of effective regurgitant orifice. First, the configuration or shape of PISA changes as the aliasing velocity changes – the convergence zone is flatter with higher aliasing velocities and becomes more elliptical with lower aliasing velocities. Secondly, the regurgitant orifice may vary during the cardiac cycle, occurring for example in the latter half of systole in MV prolapse. Colour M-mode can be used to assess variation during the cardiac cycle, but this is often not practical during stress. Thirdly, the PISA method for quantification of MR is based on the assumption that the MR jet is hemispheric proximal to the jet lesion, but this is not always the case. This is of greatest practical importance in secondary MR, when the PISA orifice may become less hemispheric and more ellipsoid, which leads to underestimation of severity. There are limited data regarding the use of pharmacological stress in MR.

**Table 1:** Standard exercise echocardiography parameters for MR assessment, with key prognostic cut-off values for primary and secondary MR.

	**Parameters**	**Key prognostic cut-off values in primary MR**	**Key prognostic cut-off values in secondary MR**
Resting quantitative assessment of disease severity	Resting BP and HR		
	EROA	Prospective 456 patients: highest survival with EROA <20 mm^2^. EROA >40 mm^2^ increases 5-year mortality rate (risk ratio 2.9) (57).	Prospective 303 patients: MR confers a graded inverse relationship with cardiac mortality (RR 1.88) (47).
	Regurgitant volume	Prospective 456 patients: adjusted mortality risk ratio increases by 1.15 per 10 mL increase in regurgitant volume (57).	
	Resting TR maximal velocity and calculation for PASP	Prospective 437 patients: resting PASP >50 mmHg is predictor of cardiovascular death (HR 2.21) (58).	
	LA volume	Prospective 492 patients: LA volume >60 mL/m^2^ reduces survival (HR 1.3), reversible with surgery (59).	
Resting left ventricular assessment	LV internal dimensions/volumes	Class I indications for surgery: LVESD ≥45 mm in ESC or LVESD ≥40 mm in AHA/ACC guidelines (5, 40).	
	Left ventricular ejection fraction	Observational 884 patients: LVEF <55% predicted mortality (28).	
	Wall motion score		
	Inferoseptal and anterolateral s′ and e′ tissue velocity	Retrospective 84 patients: resting systolic tissue velocity <10.5 cm/s predicts post-op reduction in EF (32).	
	Global longitudinal strain	Prospective 135 patients: resting GLS >−20% lowers event-free survival (33).	
Exercise parameters	Exercise BP and HR		
	Heart rate recovery post-exercise	Observational 884 patients: HRR <18 bpm/min predicts adverse events (28).	
	Duration and extent of exercise	Observational 884 patients: <100% predicted METs predicts adverse events (28).	
	Symptoms on exercise		
	Quantitative MR severity	Prospective 61 patients: EROA increase of >10 mm^2^ or regurgitant volume >15 mL predicts symptom onset (30).	Prospective 98 patients: EROA during exercise ≥13 mm^2^ associated with increased cardiac mortality (60).
	Peak TR maximal velocity and calculation for PASP	Prospective 102 patients: exercise induced PASP >60 mmHg increased risk of post-op events (31).	
	Peak LVEF		
	LVEF	Prospective 71 patients: LVEF fail to improve by ≥4% have poorer prognosis (34).	Prospective 159 patients: exercise induced PASP >60 mmHg increased rate of cardiac events (HR 5.9) (55).
	Global longitudinal strain	Prospective 71 patients: GLS fail to improve by ≥1.9% predicts post-op EF reduction (35).	

Stress echocardiography in the assessment of patients with mitral valve disease is one of the most technically demanding skills. It is expected that image acquisition requires complete training in transthoracic echocardiography, with accreditation through the British Society of Echocardiography or the reciprocal European Association of Cardiovascular Imaging credentialing. Furthermore, the individual should then have a period of supervised experience in stress echocardiography, with US recommendations for accumulation of 100 cases under supervision. The British Society of Echocardiography has introduced the first formal accreditation process in stress echocardiography that involves a written exam, acquisition of 5 cases on video for submission and a logbook of 200 cases acquired within 2 years. Candidates will also be examined while acquiring images during exercise stress. Image interpretation likewise requires extensive experience in echocardiography and those involved should have specific training.

## Summary

Although guidelines for timing of intervention in both primary and secondary MR have been established for several years, there continues to be controversy as to the appropriate timing of surgery. Quantitative exercise stress echocardiography may be a useful adjunct to the management of patients with both conditions: in primary MR, testing helps to select those asymptomatic patients with repairable valves to undergo early surgery while supporting those patients who may choose to delay intervention. In secondary MR, the role of stress echocardiography is more controversial but can help to identify the mechanism of regurgitation and target therapy in disproportionately symptomatic patients.

## Declaration of interest

The authors declare that there is no conflict of interest that could be perceived as prejudicing the impartiality of this review.

## Funding

Funding from BHF (grant no: PG/14/74/31056).
